# Can supermicrosurgery treat Alzheimer’s disease? - Current evidence and hope

**DOI:** 10.1186/s13005-026-00615-z

**Published:** 2026-04-06

**Authors:** Ashkan Rashad, Behrus Hinrichs-Puladi, Henning Wieker, Jeff Berens, Max Ulbrich, Rainer Röhrig, Frank Hölzle, Kathrin Reetz, Maurice Klein

**Affiliations:** 1 Department of Oral and Maxillofacial Surgery, University Hospital RWTH Aachen, Pauwelsstraße 30, Aachen, 52074 Germany; 2 Institute of Medical Informatics, University Hospital RWTH Aachen, Pauwelsstraße 30, Aachen, Germany; 3 Department of Oral and Maxillofacial Surgery, UKSH University Hospital Schleswig-Holstein, Arnold- Heller-Straße 3, Kiel, Germany; 4 Department of Neurology, University Hospital RWTH Aachen, Pauwelsstraße 30, Aachen, Germany

**Keywords:** Alzheimer disease, Super microsurgery, Microsurgery, Lympho-venous anastomosis, Dementia

## Abstract

Alzheimer’s disease is characterized by a loss of temporal and spatial orientation and a progressive loss of cognitive and locomotor function. Therapeutic drug options are currently limited. There is evidence that degradation products of Alzheimer’s diseases such as the tau protein or amyloid beta in Alzheimer’s disease could be removed by the glymphatic system (lymphatic system of the brain), as these drain into the deep cervical lymph nodes. The microsurgical creation of lympho-venous anastomoses could improve this drainage and improve cognition in Alzheimer’s patients (probably also for other neurodegenerative disorders). To date, however, only a few case studies and small cohort studies have been published. Despite the compelling theoretical rationale, there is currently insufficient evidence to support a therapeutic recommendation. The long-term consequences remain unknown, and a structured, stepwise program of follow-up studies is required to further investigate the proposed association. This narrative review aims to provide an overview of the opportunities as well as critical considerations in this complex field. As head and neck surgeons, we are experienced in head and neck surgery and the armamentarium of our speciality field can be widen by these procedures in the future.

## Background

### Hypothesis: can Alzheimer’s disease be influenced by super microsurgery and the arrangement of lympho-venous shunts?

**Fig. 1:**
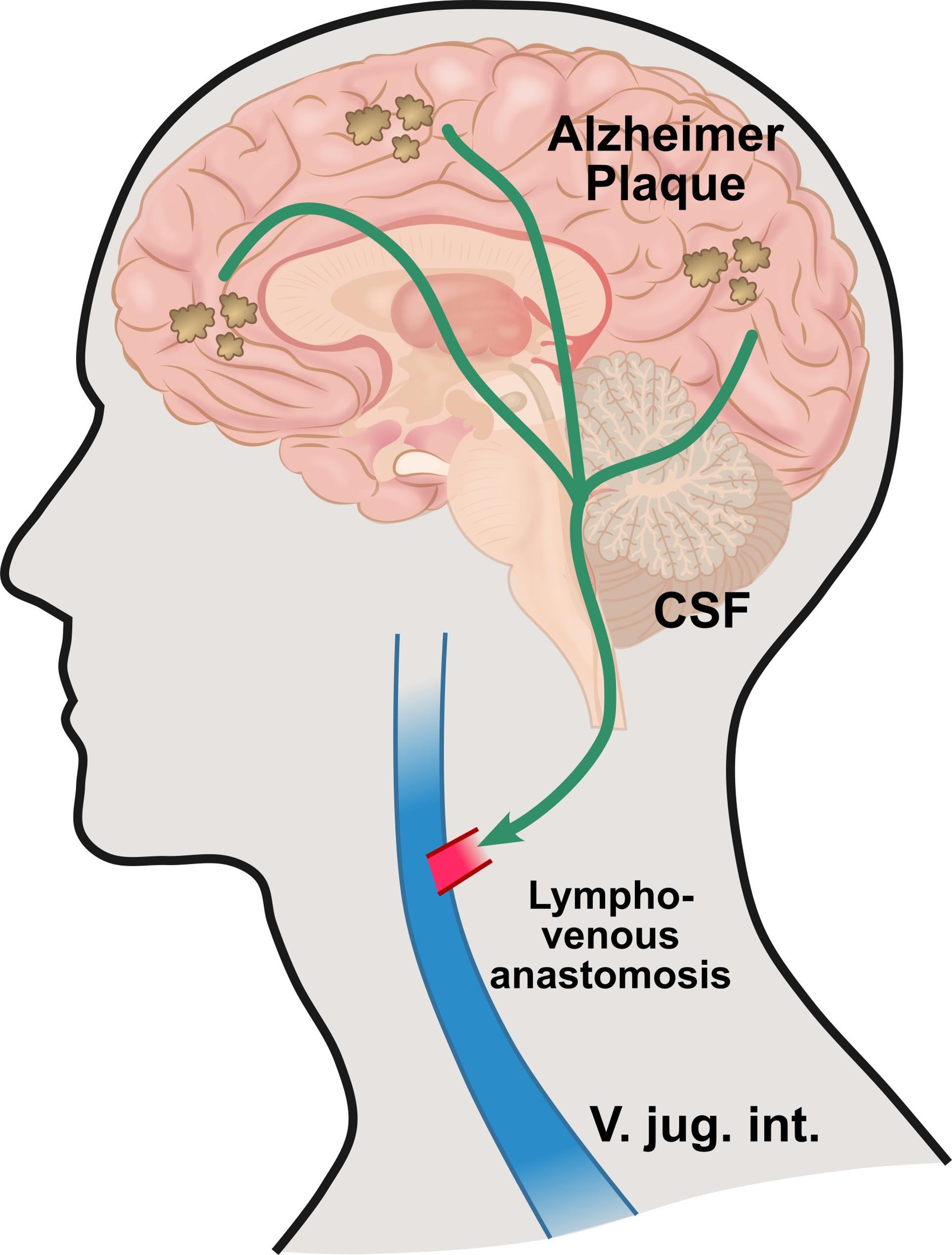
Illustration and course of degradation products of Alzheimer’s disease via cerebrospinal fluid (CSF) with hypothesized drainage improvement by microsurgical anastomosis of a cervical lympho-venous (V. jugularis interna) shunt

There are evident indications that degradation products of neurodegenerative diseases such as the tau protein or amyloid beta in Alzheimer’s disease (AD) could be removed by the glymphatic system (GS) (lymphatic system of the brain) [1]. Consecutively, the hypothesis has been put forward as to whether this removal can be supported and improved by the targeted elective microsurgical anastomosis of a lympho-venous shunt (LVS) (Fig. 1). In case report study by Li et al. even there was a rapid cognitive improvement in one patient with a follow-up of 5 weeks which seems very short and follow-up studies are required [2]. Other research groups were also able to show positive cognitive improvements through LVS in AD patients in a small group of 26 patients included. The follow-up intervals were also limited here [3]. Delaying the onset of the disease, the course of the disease or even a cure through a LVA could relieve the burden on the health and care system. This gives rise to great hope for this causally untreatable disease.

The aim of this narrative review is to provide an overview of the literature and to assess the data basis as to whether the GS could be activated by supermircrosurgery and the creation of LVA, thereby removing the pathophysiological basis of degradation products from the brain. The topic is also interesting because it could be a new and promising field of activity for head and neck surgeons.

### Alzheimer’s disease a socio-economic challenge

According to a meta-analysis, the current prevalence of AD across Europe is around 5% [4]. The economic and social impact is huge [5]. AD dementia has a high socio-economic relevance and is associated with significantly reduced survival. Survival in Europe is estimated to be around 6 years after initial diagnosis [6]. In the USA, survival rates of only 3–4 years after initial diagnosis have even been described [7].

### Alzheimer’s dementia characteristics and pathophysiological basis

Since AD is not primarily a focus of activity for the head and neck surgeon, the following is a general and basic summary of individual aspects of AD. It is important to differentiate between the general term dementia ≠ AD. The term dementia should be regarded as a symptom of an underlying disease, e.g. AD. Dementia is characterized by a cognitive loss of functions (cognitive and locomotor). In progressive cases, there is usually also a loss of orientation (time, place) and a progressive loss of participation in life [8].

The most common form of “dementia”, accounting for around 60–80% of all cases, is Alzheimer’s dementia. Vascular dementia accounts for 5–10%. However, there are also many other differential diagnoses that can make up the symptoms of dementia (Parkinson’s disease, dementia with Lewy bodies or frontotemporal dementia, mixed forms) [9].

In the following, however, the main focus will be on AD, as this also has the highest socio-economic significance. Many different risk factors for this disease have been described. In AD, for example, a high genetic predisposition is assumed, so that > 40 genetic risk loci have been described [10].

Pathophysiologically, AD is very complex and the pathophysiology of the disease has only been partially elucidated to date. What is certain, however, is that it leads to brain atrophy, amyloid plaque and tau protein deposition, neurofibrillary degeneration and immunological changes [10].

In particular, the accumulation of amyloid plaque and tau protein should be influenced by super microsurgery. In the following, the anatomy of the GS (lymphatic system of the brain) should be described in order to understand tau and amyloid plaque clearance.

### Amyloid and tau clearance with the help of the glymphatic system

Anatomic and physiologic knowledge of the GS is important for understanding amyloid and tau clearance. The GS consists of perivascular spaces that support the clearance of interstitial waste products, including amyloid-β and tau, via the cerebrospinal fluid (CSF) [1]. The CSF is then drained into the blood via cervical lymph nodes [11]. However, other studies also demonstrate this connection. Eide et al. were able to show that CSF drains tracers into cervical lymph nodes [12]. Since the GS is connected to the cervical lymphatic system, one could now, conversely, also consider the effects of the interruption of the lymphatic system on the development of AD. Chao et al. showed that dementia occurred more frequently with impaired lymphatic drainage after oncologically indicated bilateral neck dissection [13].

Unfortunately, the underlying diseases were inadequately presented and only the “symptom” of dementia (Dementia ≠ AD) was presented. In contrast, other operations also have an impact on the CSF and appear to have an influence on the incidence of AD. For example, an association of a reduction in AD was found when patients with normal pressure hydrocephalus had CSF shunts placed [14]. In summary, it can be stated that an influence from the CSF also suggests an influence on AD.

## Materials and methods

We appreciate the comment regarding the methodological framing of our work. We deliberately chose the format of a narrative review because the currently available evidence on this topic is extremely limited, heterogeneous, and largely exploratory. The existing literature primarily consists of pathophysiological considerations, preclinical data, case reports, and conceptual contributions, with no sufficiently comparable or methodologically homogeneous studies that would justify a systematic review or meta-analysis. The aim of this article is therefore not to provide a quantitative assessment of established evidence, but rather to present a structured and critical discussion of a theoretical concept, including its biological plausibility, potential risks, and existing knowledge gaps. The narrative approach allows for an integrative discussion of the interdisciplinary aspects of the topic, particularly lymphatic physiology, neurodegenerative pathomechanisms, and surgical feasibility, and supports the proposal of a cautious, stepwise research framework. The database used for the literature search was PubMed.

## Discussion

### Possible indication of lympho-venous anastomosis, diagnostics and follow-up in Alzheimer’s disease

The initial diagnosis and follow-up should be carried out by a neurologist who can rule out or diagnose any differential diagnoses. The diagnosis is confirmed according to the NIA-AA criteria (National Institute on Aging - Alzheimer’s Association) [8]. Various diagnostic tools are used for Alzheimer’s diagnostics, both pre- and postoperatively, in order to quantify cognitive changes. The first step is to test cognitive function using questionnaire tests (Mini-Mental Status, Examination Montreal Cognitive Assessment, Neuropsychiatric Inventory score). If AD has been diagnosed, patient selection and the time of surgery may be decisive for the outcome and follow-up. At present, no standardization of the indication has been defined. Patients who are already cognitively impaired or who are highly likely to develop dementia due to a genetic predisposition are possible candidates. This could have advantages in terms of prevention or delaying the onset of the disease. The longest possible follow-up > 2 years should be emphasized in order to be able to evaluate long-term effects and daily differences. The operating time should also be kept short, as the risk of post-operative delirium increases with the length of the operation [3, 15]. This must always be assessed on the basis of the postoperative cognitive evaluation. In the German dementia guideline, there are symptomatic approved drugs for Alzheimer’s therapy. On the one hand, acetylcholinesterase inhibitors (donepezil, rivastigmine, galantamine) and NMDA receptor antagonists (memantine) are approved [8]. Another interesting indication could be the surgical creation of LVA in drug-resistant patients [3]. 

In contrast, the development and European approval of Lecanemab in the early stages of AD is also promising. Lecanemab is a humanized IgG1 monoclonal antibody that binds to amyloid β. It was shown in a phase 3 study to result in cognitive improvements. In addition, it was shown that there were changes in the amyloid PET-CT compared to a placebo group [16].

There are many diagnostic possibilities. Some of these could also be used as biomarkers for therapy monitoring before and after surgery. Liquor diagnostics are recommended in the german dementia guidelines as standard diagnostic. Here, for example, Aβ42, total tau and pTau can be determined in the CSF [8, 17]. Pre- and postoperative imaging could also be used to quantify the response. Established markers are amyloid PET, 18 fluorodeoxyglucose 18FDG PET and magnetic resonance imaging [8]. In contrast, there are also current scientific efforts to develop AD blood markers. One biomarker with such potential could be the tau subtype tau 217, which could be as reliable as Liquor analysis [18]. Compared to amyloid PET, this would be an easy-to-use marker that does not cause a radiation dose. Perhaps it could be possible to assess and investigate the biochemical response and thus the effect of the LVS in the blood postoperatively. The small selection of diagnostic options presented here clearly shows the resulting interdisciplinarity of the clinical presentation of AD.

Additionally, there is evidence suggesting that assessment of the perivascular space on magnetic resonance imaging may serve as a predictor of subsequent clinical success following deep cervical LVA. However, given the small sample size (*n* = 10), further studies are required to confirm these findings [19].

It is unknown which patients could benefit from a LVA and which combination of imaging and biomarkers is suitable for monitoring the success of the therapy.

In addition, it would be important for the head and neck surgeon to identify which lymph node(s) are responsible for drainage, which are in anatomical proximity to a venous vessel and which lymph nodes and veins can be easily reached via an access. In addition, the access comorbidity of the surgical procedure should be low. Diagnosis and any hypothetical subsequent treatment of patients can only be performed within an interdisciplinary team.

### Location and type of lympho-venous anastomosis - surgical preparation

There are two conceivable options for performing LVA to improve drainage. One is intracranial and the other is extracranial placement. However, extracranial placement of the LVA should be preferred due to fewer complications and less comorbidity [2]. In general, the access should be a cosmetic result and easily accessible. Whether the anastomosis should be placed close to the brain or further caudally is unknown. The drainage effect could also depend on the localization to the venous system and the venous pressure conditions.

Lymphatic vessels form a unidirectional system that collects interstitial fluid, proteins, and immune cells from tissues and returns them to the bloodstream. Initial lymphatic capillaries have loose endothelial junctions that facilitate uptake and drain into larger collecting vessels equipped with smooth muscle cells and one-way valves, maintaining unidirectional lymph flow. Drainage is supported by intrinsic vessel contractions and extrinsic forces such as muscle activity. Lymphatic vessels play a crucial role in fluid balance, fat absorption, and immune surveillance [20]. Venous flow is driven by the pumping and suction function of the right heart. However, in the context of performing a LVA, pressure differences may alter the physiological conditions, potentially producing not only the intended effect of enhanced lymphatic clearance but also adverse consequences. The extent to which lymphatic valve function is affected or remains stable over time is unknown. Likewise, the potential harmful effects of LVA in patients with Alzheimer’s disease have not yet been determined.

It is also unknown whether a unilateral or bilateral venous anastomosis is advantageous. Which localization is advantageous is still unknown. A possible solution could be the intraoperative visualization of the skull base lymphatic drainage with the help of indocynanine green at the jugular foramen [3] This would also allow the lymph node(s) to be identified and a drain to be visualized. Further evaluation in an animal model is also warranted. Zhao et al. have described various techniques of lymphovenous anastomosis in rats [21].

### Using supermicrosurgery for lympho-venous anastomosis

Lymphatic drainage improvement techniques have long been established in plastic surgery regarding extremities [22]. In the head and neck region, there are patients with an indication for LVA for persistent head and neck lymph edema following neck dissection. It has been shown that a reduction in lymphoedema can be achieved [23]. Various techniques have been described in the literature for creating such an anastomosis [1, 24]. LVA can be performed end-to-end or end-to-side. It appears that the end-to-side anastomosis is advantageous [24]. In addition to the surgically more challenging lymph venous vessel anastomosis, a lymph node to vein anastomosis is also conceivable. Such an anastomosis with improvement of lymphatic drainage has been described for lymphatic edema of the lower extremity [22]. In contrast, Chen et al. prepared a lymphatic flap (fat-lymph node flap), which was spit from draining lymph vessels from the base of the skull. The intraoperative control of the skull base lymphatic drainage with the help of indocynanine green via the jugular foramen. The lymphatic flap was then anastomosed end-to-side to the internal jugular vein. Postoperative cognitive improvements were achieved for the AD patients [3].

The technical requirements can also be selected differently. On the one hand, a reflected light microscope and a manual anastomosis using microsurgical instruments could be selected. On the other hand, a surgical robot, e.g. Symani robot (Medical Microinstruments, Pisa Italy) or MUSA (MicroSure, Eindhoven, Netherlands) could also be used. The advantage of a robot-based system is that it eliminates hand tremors and thus appears predisposed to fine LVA [25]. The microscope and surgery robot selection can be important, as the size of the lymph vessels can vary greatly depending on the localization of the drainage area [26]. Technical developments mean that boundaries can be pushed further. In contrast, there is a possible risk of injury to the accessory nerve during the placement of LVA [3]. In this case study, the surgery was performed by neurosurgeons. Due to the significantly more frequent operations performed by head and neck surgeons and the performance of neck dissection, there is improved expertise for these neck procedures. It is not yet possible to assess the side effects or consequential complications caused by the placement of LVA in the head and neck area in combination with AD. Risks such as cognitive deterioration caused by the operation, long-term outcome and whether there is an increased tendency to thrombosis with the placement of lymphatic flap systems must also be considered.

### Uncertain therapeutic risks and the imperative for a phased research strategy

At the present time, the long-term consequences of this intervention are unknown. Importantly, potential therapy-related risks, particularly in the context of AD, cannot be adequately quantified or predicted. Any premature clinical implementation would therefore be scientifically and ethically unjustified. A rigorous, stepwise research framework is essential, beginning with comprehensive preclinical investigations to clarify mechanistic effects, hemodynamic consequences, and safety profiles, followed by carefully designed early-phase clinical trials under strict monitoring. Such a phased approach is also indispensable for systematically evaluating and comparing different anastomotic techniques with respect to safety, feasibility, and potential efficacy before considering broader clinical application. A central consideration in evaluating potential interventions for Alzheimer’s disease is the selection of appropriate clinical endpoints. In line with the ultimate goal of improving patient outcomes, the long-term trajectory of cognitive function should constitute the primary endpoint, as it most directly reflects meaningful changes in quality of life. Given the typically slow progression of the disease, follow-up periods of at least 24 months are recommended to capture clinically relevant effects. While cognitive outcomes are essential, complementary biomarker assessments may enhance the rigor of efficacy evaluation. Measures of Tau and Amyloid-β in blood or cerebrospinal fluid, as well as PET imaging, can provide mechanistic insights into disease progression and therapeutic impact. Incorporating both clinical and biomarker endpoints within a longitudinal study design would allow for a more comprehensive assessment of intervention efficacy, while also facilitating correlation between pathophysiological changes and functional outcomes. Taken together, these considerations highlight the importance of carefully designed, long-term studies in Alzheimer’s disease, with endpoints that integrate cognitive, functional, and biological measures to ensure that observed benefits translate into meaningful clinical improvements for patients. Rigorous safety monitoring is essential throughout the study to promptly identify and manage any adverse events.

## Conclusion

AD is on its way to becoming one of the most lethal, most expensive diseases of this century [9, 27]. There is hope that low-cost surgery could make an additional contribution to AD therapy. In addition, the glymphatic clearance improvement could provide a sustainable and permanent solution or improvement. Nevertheless, a conclusive demonstration of efficacy has not yet been achieved. It is important to mention that LVA for AD therapy in the head and neck region is not (yet) a standard procedure and has currently only been studied in small cohorts. In contrast, however, the available evidence is promising and requires further investigation in prospective studies. Overall, the therapeutic options for AD are currently limited. The search for additional inexpensive, sustainable and simple therapy options is therefore important. There is evidence of an effect on tau and amyloid plaques by influencing the outflow of CSF. The evidence is limited and further large-scale prospective studies are needed to investigate the effects of LVAs in AD. Nevertheless, the topic is exciting and gives hope that it could become another treatment option for AD in the future.

It is evident that these procedures should only be performed within a multidisciplinary setting (oral and maxillofacial surgery, otolaryngology, neurology, neurosurgery, and ethics) under appropriate supervision and following controlled clinical studies. Currently, the available evidence is insufficient to support routine clinical application.

Oral and maxillofacial surgeons and otorhinolaryngologists are predisposed to perform the operations due to the frequent access to the deep lymph nodes of the neck, also with regard to the frequently used microsurgical expertise. Only the future will tell whether this procedure will expand the field of activity of head and neck surgeons.

## Data Availability

No datasets were generated or analysed during the current study.

## Abbreviations

AD: Alzheimer’s disease
CSF: Cerebrospinal fluid
GS: Glymphatic system
LVS: Lympho-venous shunt
LVA: Lympho-venous anastomosis
